# Notch-1 Mediated Cardiac Protection following Embryonic and Induced Pluripotent Stem Cell Transplantation in Doxorubicin-Induced Heart Failure

**DOI:** 10.1371/journal.pone.0101024

**Published:** 2014-07-02

**Authors:** Hilda Merino, Dinender K. Singla

**Affiliations:** Biomolecular Science Center, Burnett School of Biomedical Sciences, College of Medicine, University of Central Florida, Orlando, Florida, United States of America; Georgia Regents University, United States of America

## Abstract

Doxorubicin (DOX), an effective chemotherapeutic drug used in the treatment of various cancers, is limited in its clinical applications due to cardiotoxicity. Recent studies suggest that transplanted adult stem cells inhibit DOX-induced cardiotoxicity. However, the effects of transplanted embryonic stem (ES) and induced pluripotent stem (iPS) cells are completely unknown in DOX-induced left ventricular dysfunction following myocardial infarction (MI). In brief, C57BL/6 mice were divided into five groups: Sham, DOX-MI, DOX-MI+cell culture (CC) media, DOX-MI+ES cells, and DOX-MI+iPS cells. Mice were injected with cumulative dose of 12 mg/kg of DOX and 2 weeks later, MI was induced by coronary artery ligation. Following ligation, 5×10^4^ ES or iPS cells were delivered into the peri-infarct region. At day 14 post-MI, echocardiography was performed, mice were sacrificed, and hearts were harvested for further analyses. Our data reveal apoptosis was significantly inhibited in ES and iPS cell transplanted hearts compared with respective controls (DOX-MI+ES: 0.48±0.06% and DOX-MI+iPS: 0.33±0.05% vs. DOX-MI: 1.04±0.07% and DOX-MI+CC: 0.96±0.21%; p<0.05). Furthermore, a significant increase in levels of Notch-1 (p<0.05), Hes1 (p<0.05), and pAkt (p<0.05) were observed whereas a decrease in the levels of PTEN (p<0.05), a negative regulator of Akt, was evident following stem cell transplantation. Moreover, hearts transplanted with stem cells demonstrated decreased vascular and interstitial fibrosis (p<0.05) as well as MMP-9 expression (p<0.01) compared with controls. Additionally, heart function was significantly improved (p<0.05) in both cell-transplanted groups. In conclusion, our data show that transplantation of ES and iPS cells blunt DOX-induced adverse cardiac remodeling, which is associated with improved cardiac function, and these effects are mediated by the Notch pathway.

## Introduction

Doxorubicin (DOX) is the antineoplastic drug of preference to treat a wide variety of malignancies such as acute leukemia, lymphoma and breast cancer, with a high survival rate among patients [1], [2]. However, the benefits of this drug become less appealing due to the cardiotoxic side effects after treatment [3], [4]. Moreover, some studies have found that patients receiving DOX therapy had an increased risk of myocardial infarction (MI) and that this risk persisted up to 25 years after DOX treatment [5]. DOX treatment demonstrates cardiac remodeling which results in cardiac myocyte apoptosis and fibrosis [6]–[8]. Cardiac myocyte apoptosis and heart dysfunction in DOX-induced heart failure involves many complex mechanisms including oxidative stress and upregulation of pro-apoptotic and downregulation of anti-apoptotic proteins [9]–[12].

Adjuvant therapies have been proposed to decrease the cardiotoxic effects of DOX. For instance, the use of antioxidants such as probucol, taurine, and fenofibrate have been shown to suppress DOX-induced oxidative stress and cardiac myocyte apoptosis [13]–[15]. However, none of these therapies have been shown to improve heart function at the physiological level. Since cardiac myocytes have limited self-renewal, stem cells have acquired significant consideration as an alternative method to repair and regenerate cardiac cells [16]. Of note, human umbilical cord blood derived stem cells and bone marrow-derived stem cells have been shown to improve cardiac function and capillary density in DOX-induced cardiomyopathy (DIC) [16]–[18].

Embryonic stem (ES) and induced pluripotent stem (iPS) cells have the potential to develop into cell types from all three germ layers compared with adult stem cells, which have a limited plasticity, as well as attenuate apoptosis and fibrosis [19]–[21]. Although concerns have been raised regarding the teratoma potential of these cells types, our published data as well as that of others suggest teratoma formation is dose-dependent and transplantation of up to 100,000 ES cells have not yielded teratomas up to 12 weeks post-transplantation [22], [23]. Notably, we reported recently that ES cells and their condition media (CM) inhibit apoptosis and improve cardiac function once transplanted in a DIC model [24]. However, the mechanisms by which stem cells attenuate cardiac remodeling and exert their benefits are not well understood. Therefore, we hypothesize that transplanted ES and iPS cells inhibit cardiac remodeling (apoptosis and fibrosis), mediated by the Notch pathway, and improve cardiac function in a combined model of DIC and myocardial infarction. Our data shows that transplantation of ES and iPS cells inhibits apoptosis and fibrosis in DIC following MI. Moreover, our data demonstrates that after transplantation of ES and iPS cells, levels of Notch-1, Hes1 and Akt increased significantly with decreased levels of PTEN, a negative regulator of Akt. Finally, hearts transplanted with ES and iPS cells show a significant improvement in cardiac function.

## Materials and Methods

### ES and iPS Cell Culture

CGR8 mouse ES cells were kindly provided by Michel Puceat (Centre de Researches, INSERM, U390 France) and iPS cells were generated from H9c2 cardiomyoblasts as previously described [19], both of which have been used in numerous published studies [19], [20], [24]–[26]. ES and iPS cells were maintained in Dulbecco’s Minimum Essential Medium (DMEM, Invitrogen, USA) containing leukemia inhibitory factor (LIF), sodium pyruvate, glutamine, β-mercaptoethanol, penicillin/streptomycin, nonessential amino acids and 15% ES cell-qualified fetal bovine serum on a 60 mm gelatin coated tissue culture plate as published by us [20]. Moreover, activin A and bFGF were present in the iPS growth medium but not in the ES cell medium.

### DOX Treatment

All animal protocols were approved by the University of Central Florida animal care committee as per US National Institute of Health guidelines. C57BL/6 mice, male and female, of 8–10 weeks of age, were treated with DOX as previously reported [24]. In brief, mice were injected one time every other day (Monday, Wednesday, and Friday) for three days with DOX to obtain a cumulative dose of 12 mg/kg via intraperitoneal injection (IP).

### Coronary Artery Ligation and Stem Cell Transplantation

Two weeks after the last dose of DOX, MI was induced by coronary artery ligation under isoflurane inhalatory anesthesia administered via an endo-tracheal tube as reported previously [25], [27]. In brief, a left thoracotomy was performed; the left anterior descending (LAD) coronary artery was visualized using a dissecting microscope and subsequently ligated using a 7.0 polypropylene suture. Subsequently, animals were divided into five groups: Sham (No treatment), DOX-MI, DOX-MI+cell culture (CC) media, DOX-MI+ES cells, and DOX-MI+iPS cells with an n = 6–8 in each group. For each mouse, following ligation, 5×10^4^ ES or iPS cells were delivered into two independent sites in the peri-infarct region, identified as the infarcted border zone around the LAD region where the suture was placed, using a 29-gauge floating needle. 20 µl of media was delivered in two injections of 10 µl each at two different sites in the CC group.

### Terminal Deoxynucleotidyl Transferase dUTP Nick End Labeling (TUNEL) Assay

TUNEL staining was performed as previously reported [20]. In brief, heart sections were deparafinized, rehydrated, and then incubated with proteinase K (Sigma) at a dose of 25 ug/ml in 100 mM Tris-HCL for 15 minutes. Apoptotic positive nuclei were determined by TUNEL staining according to manufacturer’s instructions. In brief, sections were incubated with a TUNEL reaction mixture for one hour, and then washed with 1×PBS three times. Finally, mounting media with DAPI was used to cover the slides. Each slide was analyzed under an Olympus fluorescent microscope. Photomicrographs were taken under 20X and the percentage of apoptotic nuclei (red) was determined by counting the total number of red positive cells and the total number of nuclei (blue). Red apoptotic nuclei that merge with DAPI in blue were considered positive. The following formula was applied to get the percentage: (red+apoptotic nuclei/total blue nuclei)*100. One to two sections of 6–8 animals per group were analyzed.

### Caspase-3 Activity Assay

The heart was homogenized, the supernatant was collected and protein quantification using a Bradford assay was performed. Caspase-3 colorimetric activity assay (Bio Vision) was performed according to the manufacturer’s instructions as previously reported [20]. In brief, the reaction buffer, provided in the kit containing 10 mM of DTT, was added to the heart homogenates. The specific enzyme, DEVD-pNA, was added to each sample and incubated for 1–2 hours at 37°C. The developed colorimetric reaction was measured at 405 nm in a 96 well microplate reader (Biorad Model) and values plotted as arbitrary units. Data collected from 4–8 animals per group.

### Notch-1 and α-Sarcomeric Actin Double Staining

The bottom part of the heart was embedded in different ethanol dilutions (75%, 80%, 90%, and 100%), and paraffin blocks were formed containing the heart tissue. Five micron sections were obtained and placed on microscope slides. A double immunofluorescent staining protocol was applied as reported previously [28]. Sections from five to six different hearts in each group were deparaffinized, rehydrated, and then blocked with a mouse antigen blocking reagent for one hour and then incubated with primary antibodies for mouse Notch-1 antibody (1∶20 dilution, abcam) and a monoclonal anti-α-sarcomeric actin antibody (1∶30 dilution; Sigma-Aldrich). Control sections were omitted for primary antibody. Biotinylated Anti-Mouse IgG reagent (MOM kit, Vector Laboratories) was applied for 1 hour at room temperature. Sections were next incubated with kit components, fluorescein Avidin DCS (16 ul/ml) or Texas Red Avidin DCS (15 ug/ml), respectively to develop the reaction. Washings with PBS were performed and sections were covered with mounting media containing 4′,6-diamino-2-phenylindole (DAPI). Fluorescence labeled cells were identified, analyzed and representative photomicrographs were taken under fluorescent (Olympus 1×70 and Nikon TE 2000-E) and confocal (LEICA laser scanning) microscopes. To get the percentage total Notch^+ve^ cells where divided to total DAPI times 100. One to two sections of 6 animals per group were analyzed.

### Sodium Dodecyl Sulfate Polyacrylamide Gel Electrophoresis and Western Blot

Western blot analysis was performed as previously reported [19], [26]. In brief, proteins were loaded in an 8% or 10% SDS-Page and electrophoresis was run at 150 V for 1 hour. Proteins were transferred to a PVDF membrane (BioRad) using a Trans-Blot Semi-dry transfer. Cell membranes were blocked with 5% skim milk in tris-buffered saline and tween 20 for 1 hour and then incubated with primary antibodies Notch-1 (Cell signaling), pPTEN (Cell Signaling), pAKT (Cell Signaling), Hes1 (Abcam) and β-actin (Cell signaling) at appropriate dilutions for 1 hour at room temperature or overnight at 4°C. Following the incubation of primary antibodies, secondary antibody was used and membranes were incubated for 1 hour. Finally, membranes were treated with an enhanced chemiluminescent substrate (ECL, Thermo Scientific) for 1–2 min and then exposed at different exposure times. β-actin was used as loading control. Blots from 4–6 different experiments were scanned and band intensities from each blot were analyzed using image J software and expressed relative to β-actin signal.

### pAkt Activity Assay

Heart homogenization and protein quantification were performed as stated before. pAkt1 (PAN) activity assay (Exalpha Biological) was performed following the directions of the manufacturer’s instructions as reported previously [24]. In brief, 50 ul of each sample was pipetted into microtiter wells that contain capture antibodies, provided by the manufacturer. Plates were incubated for two hours to develop the reaction. Washings were performed to remove any unbound material in the plates. A biotin conjugated anti-Phospho Akt1 antibody was added to each well and incubated for two hours. Excess antibody was washed and a HRP-conjugated streptavidin was added to each well for 30 min at room temperature. A colorimetric reaction was developed that was measured at 450 nm in a microplate reader. Total protein concentration of each sample was determined by Bradford assay. Values obtained from the ELISA experiments were normalized to the total protein concentration for each of the samples and were plotted as arbitrary units. Data collected from 4–5 animals per group.

### Masson’s Trichrome Staining

Heart sections were deparaffinized and rehydrated in serial dilutions of alcohol, then stained with Masson’s trichrome staining following the technique reported previously [26]. In brief, sections were incubated in Bouins Fixative for 45 min, washed and stained with Weigert Iron Hematoxylin, Anylin Blue, and finally mounted with Cytoseal-60 and cover slipped. Each slide was analyzed under a brightfield microscope with the fibrotic areas stained in blue and the healthy in red. The percentage of interstitial and vascular fibrosis area over total interstitial and vascular area was measured using Image J.

### MMP-9 Immunoassay

MMP-9 expression was determined from heart homogenates (n = 4–6 hearts/group) using an enzyme-linked immunoassay (R&D Systems) as previously described and following the manufacturer’s protocol [26]. The color reaction, proportional to MMP-9 concentration, was quantified in a microtiter plate reader at 450 nm. Results were corrected to protein concentration, which was determined by the Bradford assay. Data was plotted as arbitrary units.

### Echocardiographic Analysis

At day (D) 14 post-MI, two-dimensional (2D) echocardiography was performed under 2% isoflurane via nose cone to analyze cardiac function as previously described [24]. In brief, M-mode images of the left ventricle were documented. Left ventricular (LV) dimensions including LV fractional shortening (FS) and LV ejection fraction (EF) were measured. After echocardiography analysis was performed, animals were sacrificed using pentobarbital (80 mg/Kg) and cervical dislocation. The hearts were harvested, rinsed with PBS and sectioned transversally with the top portion kept in RNA later and the bottom portion in 10% paraformaldehyde (PFA).

### Statistical Analysis

One-way analysis of variance (ANOVA) was performed followed by Tukey test for all samples. All values were presented as a mean ± SEM. p<0.05 was considered to be statistically significant between the values.

## Results

### ES and iPS Cell Transplantation Decreases Apoptosis in DIC post-MI

**Figure 1:**
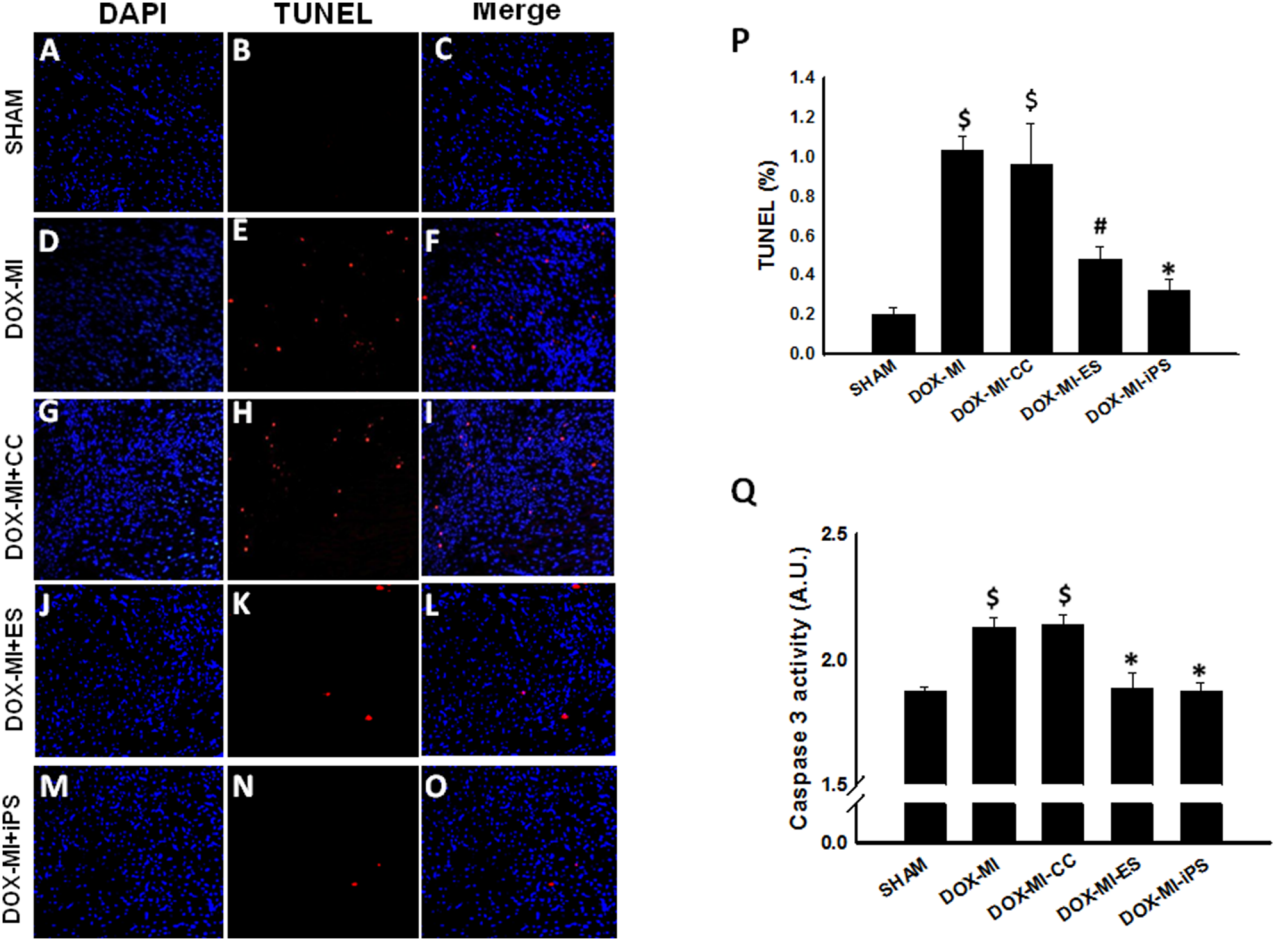
Effects of transplanted ES and iPS cells on cardiac myocyte apoptosis using TUNEL staining. Representative photomicrographs at 20X are shown on the left with DAPI in blue (**A, D, G, J and** **M**), TUNEL in red (**B, E, H, K** **and** **N**) and merged images (**C, F, I, L** **and** **O**). Top right histogram (**P**) shows the percentage of total apoptotic nuclei at 2 weeks post-MI. Bottom right histogram (**Q**) shows quantitative analysis of caspase 3 activity assay in arbitrary units. $p<0.05 vs. Sham; *p<0.05 vs. DOX-MI and DOX-MI+CC; n = 5.

To determine whether ES and iPS cell transplantation has an anti-apoptotic effect in DIC post-MI, TUNEL staining was performed. Figure 1A–O shows representative photomicrographs of TUNEL staining in each of the study groups at day 14 post-MI. After quantitative analysis, we observed a significant increase in TUNEL-positive nuclei in the DOX-MI and DOX-MI+CC groups compared with the Sham control group (p<0.001, Figure 1P). Notably, the percent apoptotic nuclei was significantly reduced in DOX-MI+ES cell and DOX-MI+iPS cell transplanted hearts compared with DOX-MI and DOX-MI+CC hearts (DOX-MI+ES: 0.48±0.06% and DOX-MI+iPS: 0.33±0.05% vs. DOX-MI: 1.04±0.07% and DOX-MI+CC: 0.96±0.21% TUNEL positive nuclei/total nuclei; p<0.05; Figure 1P). Moreover, to confirm our TUNEL results, a caspase 3 activity assay was performed using heart homogenates from each group. Statistical analysis of caspase 3 activity showed a significant increase in the activity of this apoptotic marker in DOX-MI and DOX-MI+CC groups compared with Sham (p<0.05, Figure 1Q). However, a significant decrease in caspase 3 activity in DOX-MI+ES and DOX-MI+iPS cell transplanted hearts was observed when compared with the DOX-MI and DOX-MI+CC hearts (DOX-MI+ES: 1.88±0.06 A.U. and DOX-MI+iPS: 1.87±0.03 A.U. vs. DOX-MI: 2.13±0.03 A.U. and DOX-MI+CC: 2.13±0.04 A.U.; p<0.05; Figure 1Q).

### Transplantation of ES and iPS Cells Contributes to Cardiac Repair in DIC post-MI Through Notch-1

**Figure 2:**
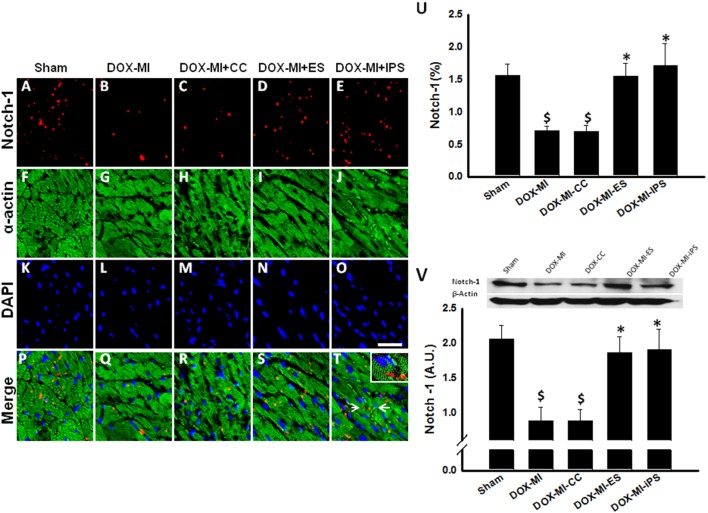
Effects of transplanted ES and iPS cells on Notch-1 expression. Representative photomicrographs are shown on the left with Notch-1 in red (**A, B, C, D and E**), α-sarcomeric actin in green (**F, G, H, I and J**), DAPI in blue (**K, L, M, N and O**) and merged images (**P, Q, R, S and T**). The white arrows (**T**) indicate the region that was magnified and shown within the boxed region. Scale bar = 20 µm. Top right histogram (**U**) shows percentage of Notch-1^+ve^ cells at 2 weeks post-MI. Bottom right panel and histogram (**V**) show representative Notch-1 blot and associate β-actin with densitometric analysis. $p<0.05 vs. Sham; *p<0.05 vs. DOX-MI and DOX-MI+CC; n = 6.

Notch-1 regulates the fate of cardiac progenitor cells (CPCs) and stimulates proliferation of cardiac myocytes [29], [30]. Previous studies have shown that after treatment with DOX, Notch-1 receptor expression levels in CPCs are low compared with its ligands, delta-like 3 and jagged [5]. Thus, we wanted to study the expression of Notch-1 in DIC-post MI hearts. Therefore, we examined Notch-1^+ve^ cells co-labeled with α-sarcomeric actin (Figure 2A–T). Quantitative analysis of immunostaining demonstrated Notch-1 receptor expression in DIC post-MI was significantly decreased in DOX-MI and DOX-MI+CC groups compared with Sham control group (p<0.05, Figure 2U). Conversely, a significant increase of Notch1^+ve^ cells in DOX-MI+ES and DOX-MI+iPS cell transplanted hearts was observed when compared with the untreated DOX-MI and DOX-MI+CC groups (DOX-MI+ES: 1.54±0.19% and DOX-MI+iPS: 1.71±0.34% vs. DOX-MI: 0.70±0.06% and DOX-MI-CC: 0.69±0.09% Notch-1^+ve^ cells/total nuclei; p<0.05; Figure 2U). Moreover, western blot (WB) analysis was performed to validate the results of our Notch-1 immunostaining. Densitometric analysis of Notch-1 bands confirmed that expression of Notch-1 in DOX-MI and DOX-MI+CC hearts was significantly decreased compared with our control Sham group (p<0.05, Figure 2V). Additionally, WB analysis also confirmed hearts transplanted with ES and iPS cells contained a significantly increased percentage of Notch-1^+ve^ cells when compared with the untreated groups, DOX-MI and DOX-MI+CC (DOX-MI+ES: 1.86±0.22 A.U. and DOX-MI+iPS: 1.90±0.29 A.U. vs. DOX-MI: 0.87±0.2 A.U. and DOX-MI+CC: 0.87±0.16 A.U.; p<0.05; Figure 2V). Statistical analysis revealed Notch-1 expression was not significant between DOX-MI+ES and DOX-MI+iPS groups.

### Effects of ES and iPS Cell Transplantation on Hes1 in DIC post-MI

**Figure 3:**
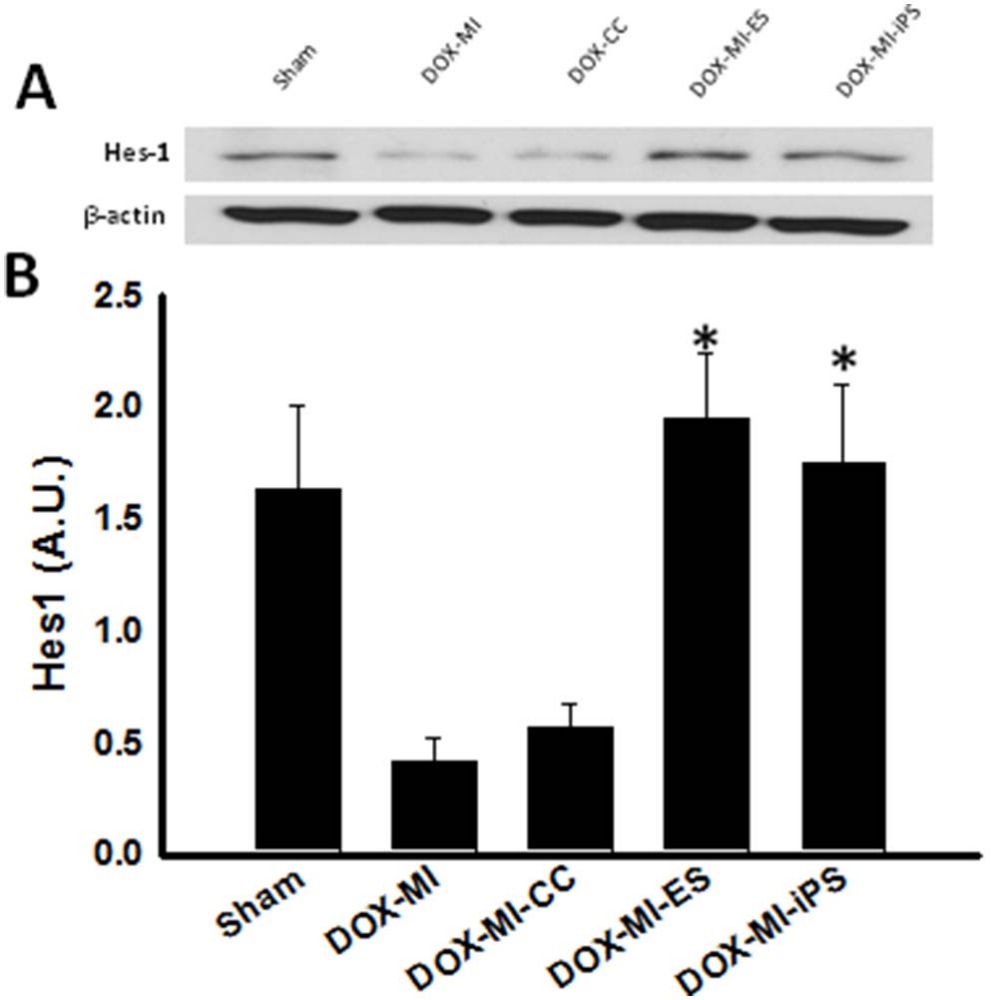
Effects of transplantation of ES and iPS cells in Hes1 in DIC post-MI. (**A**) Upper panel displays representative photomicrographs of WB bands of Hes1 and β-actin. (**B**) Bottom histogram shows densitometric analysis of WB bands with a significant increase in levels of Hes1 in groups treated with ES and iPS cells. *p<0.05 vs. DOX-MI and DOX-MI+CC; n = 5.

To further characterize the effects of Notch-1 in DIC post-MI, Hes1, a downstream effector of Notch-1, was studied. Western blot studies were performed to study the expression of this protein in DIC post-MI. Densitometric analysis of Hes1 WB bands demonstrated a significant increase in expression of Hes1 in the ES and iPS cell transplanted hearts compared with Hes1 expression in DOX-MI and DOX-MI+CC groups (DOX-MI+ES: 1.94±0.29 A.U. and DOX-MI+iPS: 1.75±0.35 A.U. vs. DOX-MI: 0.41±0.11 A.U and DOX-MI+CC: 0.56±0.10 A.U.; p<0.05; Figure 3A–B). Nevertheless, Hes1 protein levels between ES and iPS cell transplanted groups were not significant.

### Akt and PTEN Expression is Regulated by ES and iPS Cells Transplanted in DIC post-MI Hearts

**Figure 4:**
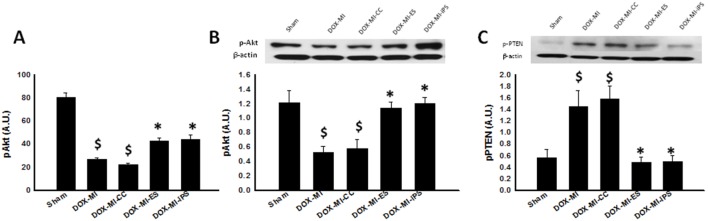
Effects of transplanted ES and iPS cells on Akt and PTEN expression in DIC post MI. Left histogram (**A**), reveals a significant increase in pAkt activity in hearts transplanted with ES and iPS cells. $p<0.001 vs. Sham; *p<0.05 vs. DOX-MI and DOX-MI+CC; n = 4–5. (**B**) Top middle panel displays representative photomicrographs of WB bands for pAkt and β-actin and bottom middle histogram shows densitometric analysis of WB bands with a significant increase in levels of pAkt levels in ES and iPS cell treated hearts. $p<0.05 vs. Sham; *p<0.05 vs. DOX-MI and DOX-MI+CC; n = 4–5. Upper right panel (**C**) shows representative WB bands of PTEN and β-actin and bottom right histogram shows densitometry analysis of WB bands with a significant decrease in levels of PTEN in hearts transplanted with ES and iPS cells. $p<0.05 vs. Sham; *p<0.05 vs. DOX-MI and DOX-MI+CC; n = 4–6.

Previous studies have shown that DOX treatment decreases expression of the pro-survival protein Akt [24]. Therefore, we studied the expression of this protein in DIC post-MI and its modulation when ES and iPS cells were transplanted. A pAkt ELISA was performed to analyze pAkt levels in DIC post-MI. pAkt activity was significantly decreased in DOX-MI and DOX-MI+CC groups compared with Sham control group (p<0.001, Figure 4A). However, increased pAkt activity was observed in hearts transplanted with ES and iPS cells compared with DOX-MI and DOX-MI+CC groups (DOX-MI+ES: 42.7±2.45 A.U. and DOX-MI+iPS: 43.87±3.84 A.U. vs. DOX-MI: 26.75±1.56 A.U and DOX-MI+CC: 22.10±1.47 A.U.; p<0.05; Figure 4A). Our ELISA data was confirmed through WB studies as shown in Figure 4B. Furthermore, our data also suggests that the increase in levels of activated Akt is due to the inhibition of PTEN, an Akt inhibitor, as demonstrated by WB analysis. Densitometric analysis of PTEN WB bands revealed a significant increase in levels of this protein in DOX-MI and DOX-MI+CC compared with Sham (p<0.05, Figure 4C). However, levels of PTEN were significantly decreased in hearts transplanted with ES and iPS cells when compared with DOX-MI and DOX-MI+CC groups (DOX-MI+ES: 0.48±0.09 A.U. and DOX-MI+iPS: 0.50±0.1 A.U. vs. DOX-MI: 1.44±0.27 A.U and DOX-MI+CC: 1.57±0.23 A.U.; p<0.05; Figure 4C).

### Effects of Transplanted ES and iPS Cells on Interstitial and Vascular Fibrosis in DIC post-MI Hearts

**Figure 5:**
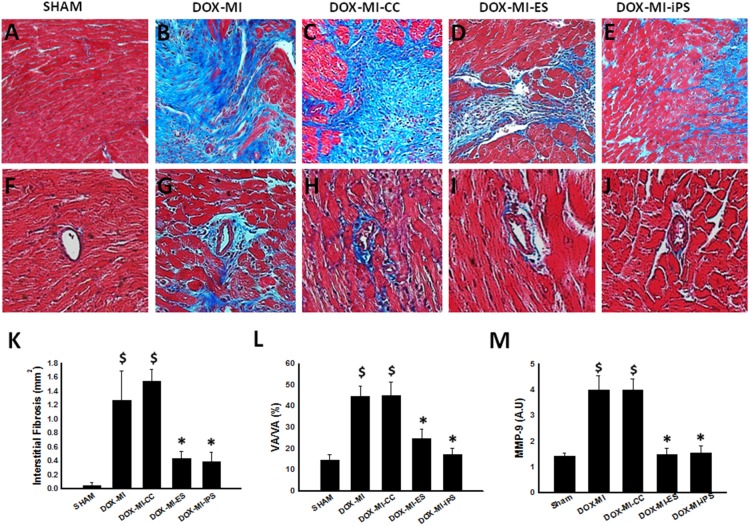
Effects of transplanted ES and iPS cells on cardiac fibrosis. Representative photomicrographs of interstitial fibrosis (**A–E**), with fibrotic tissue in blue and healthy cardiac tissue in pink and vascular fibrosis (**F–J**). Bottom left histogram (**K**) shows a significant decrease in interstitial fibrosis (mm^2^) at 2 weeks post-MI in ES and iPS cell treated groups. $p<0.05 vs. Sham; *p<0.05 vs. DOX-MI and DOX-MI+CC; n = 5–7. Bottom middle histogram (**L**) shows quantitative analysis of vascular fibrosis over total vascular area with a significant decrease in vascular fibrosis percentage in DOX-MI+ES and DOX-MI+iPS groups when compared with DOX-MI and DOX-MI+CC. $p<0.05 vs. Sham; *p<0.05 vs. DOX-MI and DOX-MI+CC; n = 4–6. Bottom right histogram (**M**) shows quantitative analysis of MMP-9 expression for all treatment groups. $p<0.01 vs. Sham; *p<0.01 vs. DOX-MI and DOX-MI+CC; n = 4–6.

To determine the effect of transplanted ES and iPS cells on interstitial cardiac fibrosis and vascular fibrosis in DIC post-MI, Masson’s trichrome staining was performed on heart sections from each of the study groups. Representative photomicrographs of Masson’s trichrome staining of interstitial fibrosis are shown in Figure 5 (A–E). Quantitative analysis performed demonstrated significant interstitial fibrosis is DOX-MI and DOX-MI+CC groups compared with the control group (p<0.05, Figure 5K). However, interstitial fibrosis was significantly decreased in DOX-MI+ES and DOX-MI+iPS cell treated hearts compared with DOX-MI and DOX-MI+CC groups (DOX-MI+ES: 0.43±0.11 mm^2^ and DOX-MI+iPS: 0.39±0.13 mm^2^ vs. DOX-MI: 1.26±0.42 mm^2^ and DOX-MI+CC: 1.54±0.16 mm^2^; p<0.05; Figure 5K). Next, quantitative analysis of vascular fibrosis in heart sections (Figure 5, F–J) revealed that DOX-MI+ES and DOX-MI+iPS cell transplanted hearts contained significantly less vascular fibrosis than DOX-MI and DOX-MI+CC groups (p<0.05, Figure 5L).

To elucidate mechanisms by which ES and iPS cells propagate fibrosis inhibition in DIC post-MI myocardium, MMP-9, a mediator of extracellular matrix (ECM) degradation and subsequent fibrosis, was quantified. MMP-9 expression was significantly enhanced in the DOX-MI and DOX-MI+CC groups compared to the Sham control group (p<0.01; Figure 5M). Notably, MMP-9 concentration was significantly reduced following ES and iPS cell transplantation (DOX-MI+ES: 1.48±0.25 A.U. and DOX-MI+iPS: 1.55±0.26 A.U. vs. DOX-MI: 3.97±0.56 A.U and DOX-MI+CC: 3.97±0.44 A.U.; p<0.01; Figure 5M).

### ES and iPS Cell Delivery in DIC post-MI Model Improves Cardiac Function

**Figure 6:**
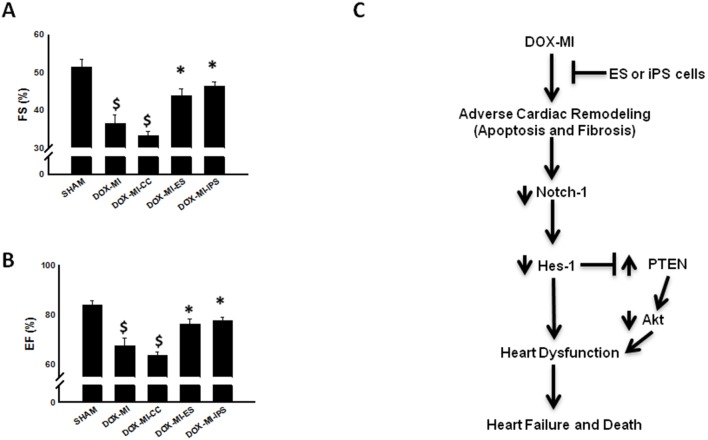
Effects of transplanted ES and iPS cells on cardiac function. Left top (**A**) histogram show that average left ventricular fractional shortening percentage (FS) significantly improved 2 weeks post-MI in the ES and iPS cell treated groups when compared with DOX-MI and DOX-MI+CC. $p<0.001 vs. Sham; *p<0.05 vs. DOX-MI and DOX-MI+CC. Left bottom (**B**) histogram show that average left ventricular ejection fraction percentage (EF) significantly improved 2 weeks post-MI in the ES and iPS cell treated groups compared with DOX-MI and DOX-MI+CC groups. $p<0.001 vs. Sham; *p<0.05 vs. DOX-MI and DOX-MI+CC; n = 4–8 animals. Right diagram (**C**) is a representation of the predicted pathway by which stem cells contribute to cardioprotection and improved left ventricular function in the DIC post-MI heart.

Two weeks following MI surgery, cardiac function was analyzed using 2D echocardiography. Our data demonstrates a significant impairment of left ventricle (LV) function expressed as fractional shortening (FS) and ejection fraction (EF) in DOX-MI and DOX-MI+CC groups compared to the Sham control group (p<0.001; Figure 6A–B). Cumulative quantitative data revealed a significant improvement in fractional shortening at 2 weeks post MI in DOX-MI+ES and DOX-MI+iPS cell treated hearts compared with DOX-MI and DOX-MI+CC groups (p<0.05; Figure 6A). Finally, DOX-MI+ES and DOX-MI+iPS transplanted hearts had a significant improvement in ejection fraction compared with DOX-MI and DOX-MI+CC groups (DOX-MI+ES: 76.43±1.88% and DOX-MI+iPS: 77.78±1.39% vs. DOX-MI: 67.42±3.30% and DOX-MI+CC: 63.51±1.63%; p<0.05; Figure 6B).

## Discussion

Doxorubicin is one of the most conventionally used anthracycline drug currently on the market for the treatment of various neoplastic diseases. Although its efficacy is widely accepted in the clinical arena in the aforementioned capacity, DOX has been shown to have dose-dependent deleterious effects on intrinsic cardiac architecture and function; these cardiotoxic consequences to include promotion of cardiac myocyte apoptosis, hypertrophy, enhanced susceptibility to MI, dilated cardiomyopathy, and impaired ejection fraction [5]–[8]. Limited studies have provided insight into the salutary impact propagated by transplanted stem cells in the DOX injured myocardium but, to date, no studies have been undertaken to elicit the effects of cellular therapy in the DOX-induced post-MI heart [16], [18], [24], [31]. In the present study, we have generated a DIC post infarction mouse model and evaluated the response of the injured myocardium to the transplanted ES and iPS cells as well as identified signaling molecules, including Notch-1, Hes1, PTEN, and Akt, which play a pivotal role in the cytoprotective mechanisms conferred by our transplanted stem cells. To the best of our knowledge, this is the first investigation into the cytoprotective impact of iPS and ES cells in the DIC post-MI injured myocardium.

Previously suggested, apoptosis plays a monumental role in cardiac myocyte cell death in DIC and post-MI hearts contributing to hypertrophy, fibrosis, diminished cardiac function, and heart failure [20], [32], [33]. Consistent with these earlier studies, our disease mouse model (DOX-MI and DOX-MI+CC) contained significantly elevated apoptotic nuclei relative to the sham operated mice as evidenced by TUNEL staining and a caspase-3 activity assay. Our data further suggests that following stem cell transplantation in the DIC post-MI heart, apoptosis is significantly attenuated. Our findings are in accordance with previous independent investigations in which they demonstrated transplanted stem cells alleviate DIC and post-MI cardiac myocyte apoptosis [16], [20], [24].

Functional characteristics of the Notch pathway in the heart include differentiation, cardiac myocyte expansion, valve formation, and cardioprotection during assault [34]–[36]. Inversely, ventricular septal anomalies, valve aberrations, and exacerbated hypertrophy and apoptosis have been reported as a result of Notch dysregulation [36]–[38]. Recently, De Angelis et al reported Notch-1 expression was significantly downregulated in CPCs following DOX treatment [1]. To this end, we evaluated changes in Notch-1 expression consequent to DIC post-MI induction and stem cell transplantation. In the control treatment groups (DOX-MI and DOX-MI+CC), a significant reduction in Notch-1 expression was observed compared to the sham controls. Conversely, a previous study reported increased levels of Notch-1 in the border zone of the infarct region post permanent coronary ligation [29]. We hypothesize variances in the experimental design including different animal models and our use of DOX account for the discrepancies reported in the expression levels of Notch-1 post-MI [29]. To the best of our knowledge, we are the first to show that expression of Notch-1 is significantly diminished in DIC post-MI hearts and suggest its dysregulation plays a role in post DOX-MI cardioprotection loss. Notably, we report a significant elevation in Notch-1 expression in both stem cell transplanted groups compared to the DIC post-MI control hearts. We suggest the enhanced Notch-1 expression observed within the ES and iPS cell transplanted DIC post-MI myocardium is consequent to paracrine mechanisms mediated by the stem cells.

To further elucidate the involvement of Notch-1 in cardiac repair and protection in the DIC post-MI heart, Hes1, a Notch-1 downstream target gene, was also investigated. Activation of Hes1 is an indirect response to activation of Notch by Delta/Jagged ligands and leads to downregulation of various targets [29], [39]. Our data implicates a dramatic increase in Hes1 expression following ES and iPS cell transplantation into the DIC post-MI heart compared to the DOX-MI and DOX-MI+CC groups. The pathway by which ES and iPS cells, transplanted in the DIC post-MI heart, promote activation of Notch-1 and its downstream effectors is largely unknown and will require further investigations.

Recent studies have suggested an interaction between the Notch pathway and the pro-survival PI3K/Akt pathway [39]. Specifically, Hes1 was shown to negatively regulate PTEN, an inhibitor of PI3K/Akt signaling, in thymocytes and T-cell lymphoblastic leukemia (T-ALL) cells [39], [40]. Our data corroborates these previous findings and suggests an inverse relationship between activation of pAkt and inhibition of PTEN expression in our study groups. In the DOX-MI and DOX-MI+CC groups, p-Akt expression was significantly downregulated and PTEN significantly upregulated comparable to the sham control group. Conversely, when DIC post-MI hearts were transplanted with ES or iPS cells, levels of Akt were dramatically elevated and levels of PTEN were drastically diminished relative to controls (DOX-MI and DOX-MI+CC). It is feasible to suggest that upregulation of Notch-1 and subsequent activation of Hes1 lead to abrogation of PTEN transcription, thus preventing the inhibition of Akt activation.

Fibrosis plays a major role in adverse cardiac remodeling in DIC and post-MI myocardium. As expected, DIC post-MI hearts without stem cell transplantation presented elevated quantities of interstitial and vascular fibrosis. On the contrary, hearts transplanted with ES or iPS cells showed significant reduction in the amount of both interstitial and vascular fibrosis. Supporting evidence of our findings include previously published articles demonstrating decreased fibrosis post stem cell transplantation in DIC and infarcted myocardium and Notch regulation of fibrogenesis [20], [24], [36]. Furthermore, MMP-9, a well-recognized mediator of adverse ventricular fibrosis and subsequent remodeling, was evaluated to establish a relationship between ES and iPS cells and blunted fibrosis in the DIC post-MI myocardium. Consistent with previous studies, transplantation of ES and iPS cells significantly abolished MMP-9 activation suggesting mechanisms of fibrosis inhibition within the current study may be similar to those reported in other MI and DOX studies [41]–[43]. However, future studies are needed to identify augmented fibrotic pathways within the ES and iPS cell transplanted DIC post-MI heart.

Finally, we needed to determine the effects of transplanted ES and iPS cells on overall cardiac function in the DIC post-MI myocardium. DIC and post-MI myocardium contribute to anomalous left ventricular stiffness and systolic dysfunction [4], [20], [24]. Within the current study, we demonstrate poor cardiac function in DOX-MI and DOX-MI+CC mice. However, DIC post-MI mice receiving stem cell transplantation exhibited significantly improved fractional shortening and ejection fraction compared to their non-stem-cell-transplanted DIC post-MI counterparts. We acknowledge that mechanisms leading to improved cardiac function are complex and multifaceted. We do however suggest that the reduction in apoptosis and fibrosis is directly related to the improvement in ventricular function observed within the present study. Conceivably, it is possible to note that cardiac function modulation may be attributable to the cardioprotective effects of the Notch pathway activation (Figure 6, C).

Stem cell transplantation, most notably ES cells, coincides with concerns of teratoma formation. In the current study, we report no evidence of teratoma formation in hearts transplanted with iPS or ES cells (data not shown). Our findings are in agreement with various published investigations indicating no teratoma formation following transplantation of less than 300,000 stem cells [20], [26]. Furthermore, our DIC post-MI model exhibited no indication of immune rejection following stem cell transplantation as reported previously by us and others [24], [44].

In conclusion, the major findings of the present study include the following for the first time: In the DIC post-MI heart, following ES and iPS cell transplantation, (1) apoptosis was significantly inhibited, (2) Notch-1 and Hes-1 expression were significantly increased, (3) fibrosis and MMP-9 expression were significantly diminished, (4) Akt activation was significantly enhanced and PTEN levels were abolished, and (5) cardiac dysfunction was mitigated. We would like to point out that our findings suggest transplanted iPS and ES cells have similar cytoprotective potential within the DIC post-MI myocardium. Furthermore, our findings are novel and, for the first time, provide evidence indicating the Notch pathway plays a role in DIC post-MI cardioprotection. However, future studies are warranted, using specific inhibitors of the Notch and Akt pathways, to understand the exact mechanisms by which ES and iPS cells protect the DIC post-MI myocardium from apoptosis and fibrosis.
